# Electrochemical Noise Analysis: An Approach to the Effectivity of Each Method in Different Materials

**DOI:** 10.3390/ma17164013

**Published:** 2024-08-12

**Authors:** Jesús Manuel Jáquez-Muñoz, Citlalli Gaona-Tiburcio, Ce Tochtli Méndez-Ramírez, Cynthia Martínez-Ramos, Miguel Angel Baltazar-Zamora, Griselda Santiago-Hurtado, Francisco Estupinan-Lopez, Laura Landa-Ruiz, Demetrio Nieves-Mendoza, Facundo Almeraya-Calderon

**Affiliations:** 1 Universidad Autónoma de Ciudad Juárez, Ciudad Juárez 32315, Mexico; jesus.jaquez@uacj.mx; 2 Centro de Investigación e Innovación en Ingeniería Aeronáutica (CIIIA), Universidad Autónoma de Nuevo León FIME, San Nicolás de los Garza 66455, Mexicofrancisco.estupinanlp@uanl.edu.mx (F.E.-L.); facundo.almerayacld@uanl.edu.mx (F.A.-C.); 3 Facultad de Ingeniería Civil, Universidad Veracruzana, Xalapa 91000, Mexico; mbaltazar@uv.mx (M.A.B.-Z.); lalanda@uv.mx (L.L.-R.); dnieves@uv.mx (D.N.-M.); 4 Facultad de Ingeniería Civil, Universidad Autónoma de Coahuila, Torreón 27276, Mexico; santiagog@uadec.edu.mx

**Keywords:** electrochemical noise, statistical analysis, power spectral density (PSD), wavelets, Hilbert–Huang transform (HHT), recurrence plots (RPs)

## Abstract

Corrosion deterioration of materials is a major problem affecting economic, safety, and logistical issues, especially in the aeronautical sector. Detecting the correct corrosion type in metal alloys is very important to know how to mitigate the corrosion problem. Electrochemical noise (EN) is a corrosion technique used to characterize the behavior of different alloys and determine the type of corrosion in a system. The objective of this research is to characterize by EN technique different aeronautical alloys (Al, Ti, steels, and superalloys) using different analysis methods such as time domain (visual analysis, statistical), frequency domain (power spectral density (PSD)), and frequency–time domain (wavelet decomposition, Hilbert Huang analysis, and recurrence plots (RP)) related to the corrosion process. Optical microscopy (OM) is used to observe the surface of the tested samples. The alloys were exposed to 3.5 wt.% NaCl and H_2_SO_4_ solutions at room temperature. The results indicate that HHT and recurrence plots are the best options for determining the corrosion type compared with the other methods due to their ability to analyze dynamic and chaotic systems, such as corrosion. Corrosion processes such as passivation and localized corrosion can be differentiated when analyzed using HHT and RP methods when a passive system presents values of determinism between 0.5 and 0.8. Also, to differentiate the passive system from the localized system, it is necessary to see the recurrence plot due to the similarity of the determinism value. Noise impedance (Z_n_) is one of the best options for determining the corrosion kinetics of one system, showing that Ti CP2 and Ti-6Al-4V presented 742,824 and 939,575 Ω·cm^2^, while R_n_ presented 271,851 and 325,751 Ω·cm^2^, being the highest when exposed to H_2_SO_4_.

## 1. Introduction

Different conventional electrochemical techniques have been used to determine the corrosion kinetics and reaction mechanisms, such as potentiodynamic polarization (PP), electrochemical impedance spectroscopy (EIS), and linear polarization resistance (LPR). However, these techniques can alter the electrochemical system with external signals in electrochemical measurements [1,2,3,4,5,6]. Using the electrochemical noise (EN) technique for investigation and corrosion monitoring has allowed many advances in recent years in corrosion science. A particular advantage of EN measurements is that they detect and analyze the early stages of localized corrosion.

Electrochemical noise describes the spontaneous low-level potential and current fluctuations during an electrochemical process. The EN can be used to monitor different corrosion processes, but it is specialized in localized processes; the type of analysis is linked to the type of signal present in the system. One advantage of EN is its efficiency in localized processes, which is a non-perturbative technique [7,8,9,10,11].

The EN analysis can be classified into time-domain, frequency-domain, frequency–time, and chaotic systems. In the first years of the technique, the signal was analyzed by the visual method; the next analysis realized the EN signal by realizing the statistical method, where authors such as Mansfeld, Cottis, Turgosse, Eden, and Bertocci [11,12,13,14,15,16,17,18] related the type of corrosion with statistical parameters such as localization index (LI) and pitting index (based on the standard deviation of ECN and EPN). Furthermore, the noise resistance (R_n_) parameter was obtained and is employed as a homolog of R_p_, relating to a kinetic variable. Furthermore, some authors [15,16,17,18] used kurtosis and skewness to improve the LI to determine the corrosion type.

The components that make up the EN signal include DC, random, and stationary. DC must be separated from stationary and random components to analyze EN data. DC introduces false frequencies and interferes with statistical, visual, and PSD assessments. This way, corrosion data presented at low frequencies are maintained when DC is removed. EN can be expressed via Equation (1) [15,19,20]:

(1) $$x\left(t\right)=m_{t}+s_{t}+Y_{t}$$

The noisy signal (*x_n_*) and polynomial of “*n*” grade (*p_o_*) at *n*-th term (*a_i_*) in “*n*” time are defined by the polynomial approach, as stated in Equation (2), to produce a signal devoid of trend (*y_n_*) [1,11,21,22]:

(2) $$y_{n}=x_{n}-\sum_{i=0}^{p_{o}}a_{i}n^{i}$$

To determine noise resistance (R*_n_*), obtaining a standard deviation from time series values (EPN and ECN) is necessary. See Equation (3); these statistical values give information about corrosion kinetics and mechanisms. Turgoose and Cottis [11] found a relationship between the increase in variance and standard deviation and an increase in corrosion rate.

(3) $$R_{n}=\frac{\sigma_{v}}{\sigma_{I}}*A$$

Kurtosis and skewness were used in this study to identify the type of corrosion. It was not considered because Mansfeld and Sun [22] decided in 1995 that the localization index (L.I.) exhibits limits and should be used with prudence. According to a patent developed in 2001 by Reid and Eden [23], corrosion type can be identified using statistical moments with skewness and kurtosis (Equations (4) and (5)), which are the third and fourth statistical moments, respectively [24,25,26,27,28]:

(4) $$skewness=\frac{1}{N}\sum_{i=1}^{N}\frac{{{(x}_{i}-\bar{x})}^{3}}{\sigma^{3}}$$

(5) $$skewness=\frac{1}{N}\sum_{i=1}^{N}\frac{{{(x}_{i}-\bar{x})}^{3}}{\sigma^{3}}$$

For PSD analysis, it is necessary to transform the time-domain EN to the frequency-domain by applying a fast Fourier transform (FFT). Since there is a correlation with the EN signal (with a polynomial filter applied), the spectral density is calculated with Equations (6) and (7) [29,30].

(6) $$R_{xx}\left(m\right)=\frac{1}{N}\sum_{n=0}^{N-m-1}x\left(n\right)\cdot x\left(n+m\right)\;when\;values\;are\;from\;0<m<N$$

(7) $$\Psi_{x}\left(k\right)=\frac{\gamma \cdot t_{m}}{N}\cdot \sum_{n=1}^{N}\left(x_{n}-{\bar{x}}_{n}\right)\cdot e^{\frac{{-2\pi kn}^{2}}{N}}$$

The PSD is interpreted using the limit frequency to cut frequency as a basis. The cut frequency indicates the start and end of the slope, which is useful in determining the corrosion mechanism. Information regarding sample representation following pitting is provided by cut frequency [31]. The slope is represented by Equation (11) and is defined by *β_x_*:

(8) $$log\;\Psi_{x}=-\beta_{x}\log f$$

Because the power PSD is correlated with the overall amount of energy in the system, the frequency zero limit (ψ^0^) provides information on material disintegration [32]. Material dissolution is limited to the current PSD [33,34]. In 1998, Mansfeld et al. [35] suggested determining the corrosion phenomena on the material surface using the intervals of *β* (adjusted to decibels) [36,37]. It is important to emphasize that some values are the same for two types of corrosion; this could create another way of studying the slope along frequencies [38,39].

In trying to develop the analysis, different authors used the wavelet transform to make a signal decomposition generating an energy plot, where the signal is divided on crystal, usually from 1 to 8. The authors related the first crystal energy, D1 to D3, to metastable pitting. The crystals D4 to D6 are associated with localized corrosion, while D7 and D8 are linked to diffusion or controlled processes (uniform corrosion) [40]. The S8 crystal is associated with the DC signal from the EN signal [15,16,18,41]. With wavelets, a signal is broken down using a high-low filter; high frequencies are referred to as detail, and low frequencies are approximations [42]. Equation (9) [43] provides the total energy of an N number of data points.

(9) $$E=\sum_{n-1}^{N}x_{n}^{2}$$

Additionally, Equation (10) provides the energy fractions of details and approximations:

(10) $${ED}_{j}^{d}=\frac{1}{E}\sum_{n=1}^{N}d_{j,n}^{2}\;{ED}_{j}^{s}=\frac{1}{E}\sum_{n=1}^{N}s_{j,n}^{2}$$

Equation (11) states that the total energy evaluated is equal to the energy of each wavelet transform component:

(11) $$E={ED}_{j}^{s}\sum_{j=1}^{j}{ED}_{j}^{d}$$

Another type of analysis is the time-frequency domain, which is presented by Hilbert–Huang transform (HHT) [31]. This method is based on an empirical decomposition (EMD) that permits analysis of the non-stationary signal obtained from the intrinsic function of the signal. The graphic obtained is the Hilbert specter, which helps to determine the corrosion process that occurs in the system and the corrosion mechanism; authors such as Mol, Zhao, and Homborg [30,31,39,40] suggest that the energy accumulated at low frequencies is related to uniform and diffusion processes, but if the energy is accumulated at middle and high frequencies, it is related to localized processes.

The HHT is an additional sophisticated technique for identifying the kind and process of corrosion; it assists in eliminating DC from the original signal [43]. Furthermore, using the HHT suggested by Huang et al. [44] in 1998 to investigate non-stationary signals, this method can also localize the frequency and time at which energy interchange occurs. This energy is known as instantaneous energy and is calculated by an empirical method of decomposition (EMD) to obtain intrinsic functions (IMF). It is possible to localize the collected energy by generating a spectrum with the time-frequency-energy distribution [45,46]. Huang’s proposed EMD is represented by Equation (12):

(12) $$x\left(t\right)=\sum_{i=1}^{N}h^{\left(i\right)}\left(t\right)+d(t)$$

where *h*^(*i*)^(*_t_*) is the *i*-th term of the IMF that is generated; these numbers must satisfy that the extreme and cross numbers are equal or differ in one at maximum and that each point using the local maximum and minimum must be zero [47]. *d*(*t*) is the average trend at a low frequency in the time series *x*(*t*) and cannot be decomposed. Equation (13) of HHT is controlled by the following:

(13) $$y_{j}\left(t\right)=\frac{1}{\pi }p\int_{-\infty }^{\infty }\frac{h_{j}(\tau )}{t-\tau }d\tau$$

The Hilbert transform is denoted by *y_j_*(*t*), and the IMF is represented by *h_j_*; *p* is associated with the Cauchy principle and the IMF average [48].

One of the methods to analyze a non-linear (chaotic) system can be interpreted by recurrence plots (RPs). RPs help distinguish if the processes in the system are deterministic recurrence processes. If the system is deterministic (D), it is related to localized processes, and if the recurrence is the system’s domain, the process is associated with uniformity [49,50]. A two-dimensional binary diagram known as a recurrence plot (RP) encodes the temporal pattern of a single recorded time series of an observable, such as the current I in this study. It depicts recurrences of a trajectory *x_i_* ∈ Rm at distinct periods *i*, *j*, that occur in m-dimensional phase space and within a given threshold limit *ε*. Specifically, an RP is a picture of a two-dimensional square matrix with black and white dots on two axes of time, *t_i_* and *t_j_*, representing ones and zeros, respectively. Each black dot at a position (*t_i_*, *t_j_*) denotes the state *x*(*t_i_*) reoccurring at time j. The mathematical expression (Equation (14)) populates the matrix [51].

(14) $$R_{ij}=\Theta \left(\varepsilon -\left\|\vec{x_{i}}-\vec{x_{j}}\right\|\right),\;i,\;j=1,\ldots ,\;N$$

where *N* is the number of measured points in this case. *x_i_*, epsilon represents the threshold distance, *θ_x_* denotes the heavy function, and ∥.∥ is normal and can be either Euclidian or maximum. The threshold epsilon constraint is responsible for the binary black-and-white appearance of recurrence graphs and for enabling the quantification of certain values [52].

Authors Ren et al. [53] conducted research to identify corrosion types in electrochemical noise; they used an Adaboost system based on statistical analysis and shot noise theory, and they found that it is possible to identify the corrosion type based on shot noise theory and statistical results, with the shot noise being the complement for statistical results. Montoya-Rangel et al. [54] employed the EN to determine the corrosion mechanism in DP steels. When exposed to chlorides, the PSD analysis detected a galvanic couple in the steel phases. Conventional EN methods cannot obtain that result. Ye et al. [55] did similar research; however, they employed the Wavelets method to determine the galvanic corrosion that occurs on DSS 2205, obtaining good results for this analysis, which was more precise than other methods. The wavets method is used to analyze more sophisticated systems, as Shahri et al. [56] analyzed localized corrosion of PEO coatings. The EN technique analyzed using the wavelet method helped to determine the localized corrosion system and its weaknesses. Homborg et al. [57] realized the importance of employing EN in different systems, such as heat treatment, corrosion inhibition, and the behavior of alloys with different phases. Also, it mentioned the importance of DC drift from the original signal. They concluded that the transient analysis can be treated as an image classification problem and that a wavelet transform helps determine the corrosion system.

Also, the authors work on using HHT to determine processes in dynamic systems, and stochastic behavior is one of the most important limitations of other EN methods. The ability to detect spontaneous reactions is an important advantage over other methods, as is the regeneration of some passive layers [58,59,60]. Ortíz-Corona et al. [61] employed recurrence plots to determine the chemical reactions that occur in Ag–Cu alloys, and they concluded that in the time series, the transitions of corrosion types could not be distinguished by statistical methods, and with the RPs analysis, the different transitions that occur in the system can be observed, obtaining a better performance than statistical analysis. The use of RPs is highly important in non-linear systems, according to different authors, due to the precision and diversity of results that can be analyzed because it does not present limitations for some signals [62,63,64].

This research aimed to study, employing electrochemical noise, the effectiveness of each analysis method in different aeronautical alloys (AISI 1018 CS, 304 SS, 316 SS, Inconel 718, Al 2024, AA 2055, AA 6061, Custom 450, Ti-6Al-4V, Ti CP2, Ultimet, and Waspaloy) related to the corrosion process. Optical microscopy (OM) is used to observe the surface of the tested samples. The alloys were exposed to 3.5 wt.% NaCl and H_2_SO_4_ solutions at room temperature. Electrochemical characterization of these alloys could find potential in aeronautical applications such as fuselage, turbine blades, aircraft landing gear, and structural components. The alloys of aircraft are susceptible to localized or general corrosion when they are exposed to different atmospheres: industrial [acid rain (H_2_SO_4_)] and marine (NaCl).

## 2. Materials and Methods

### 2.1. Materials

The materials used in this work were aeronautical alloys AISI 1018 CS, 304 SS (Austenitic), 316 SS (Austenitic), Inconel 718 (Nickel-based), AA 2024 (Aluminum-Copper), AA 2055 (Aluminum-Lithium), AA 6061, AM 350 (semi-austenitic), Custom 450 (Martensitic), Ti-6Al-4V, Ti CP2 (Pure Titanium), Ultimet (Cobalt-based), and Waspaloy (Nickel-based), used in the received condition.

### 2.2. Microstructural Characterization

The sample preparation was realized using metallographic techniques according to ASTM E3 [65]. The polishing was performed using different SiC grit papers until 600 grades, followed by ultrasonic cleaning in ethanol (C_2_H_5_OH) and deionized water for 10 min each.

The microstructural analysis was carried out by optical microscopy (OM, Olympus, Hamburg, Germany) to identify samples’ morphology at a magnification of 100×.

### 2.3. Electrochemical Testing

**Figure 1:**
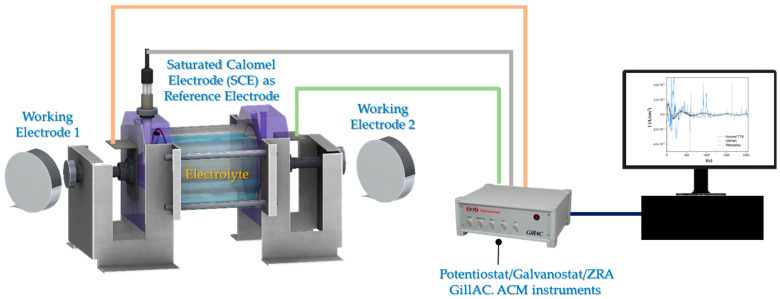
Experimental setup for electrochemical noise (EN) measurements.

The electrochemical measurements were made at room temperature using a conventional three-electrode cell (Figure 1). The working (aeronautical alloys) and auxiliary electrodes were similar electrodes, and a saturated calomel electrode was used as reference [66,67,68,69]. EN measurements were carried out according to the ASTM G199-09 standard [66]. In each experiment, 2048 data points were measured with a scanning speed of 1 data/s. The current and potential time series were visually analyzed to interpret the signal transients and define the behavior of the frequency and amplitude of fluctuations as a function of time. The electrolytes used were 3.5 wt.% NaCl and H_2_SO_4_ solutions. Tests were performed in duplicate. The electrochemical noise measurements were recorded simultaneously using a Gill-AC potentiostat/galvanostat/ZRA (Zero Resistance Ammeter) from ACM Instruments (Manchester, UK). The tests were realized in triplicate.

Data analysis was processed using a program made in MATALB 2018a software (Math Works, Natick, MA, USA). In the time-domain analysis, the DC trend signal was removed from the original EN signal by the polynomial method, and from the signal without DC, statistical data (R_n_, kurtosis, and skewness) were obtained. For frequency-domain analysis of PSD (power spectral density) data, a Hann window was applied before being transformed to the frequency domain by an FFT (fast Fourier transform). Frequency–time-domain analysis energy dispersion graphs were made (EDP), where the orthogonal wavelet transform was applied to the original signal (with DC) because this method separates DC from EN signal. EN analysis with Hilbert–Huang transform (HHT) was necessary to obtain the intrinsic functions (IMF) of EN signal by an empirical decomposition method (EMD), and finally, the instantaneous frequencies were plotted with a Hilbert spectrum.

## 3. Results and Discussion

### 3.1. Electrochemical Noise (EN)

#### 3.1.1. Time-Domain Analysis

**Figure 2:**
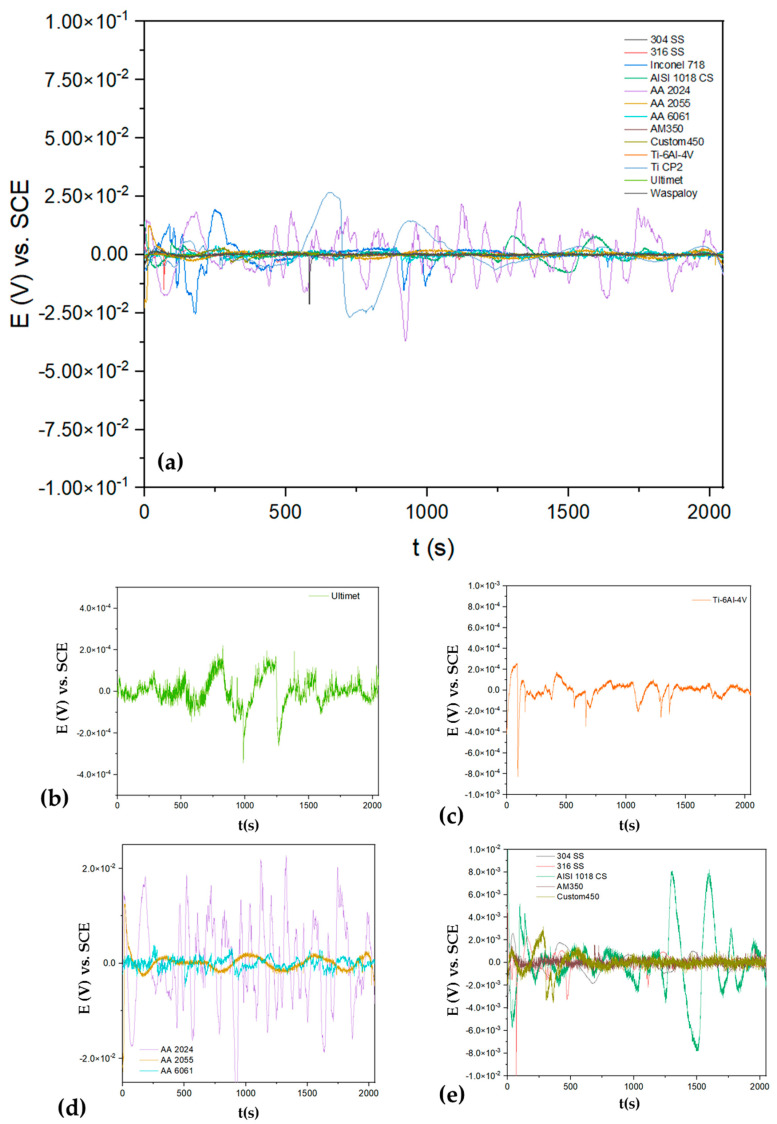
Electrochemical potential noise-time series for alloys in NaCl (**a**) and windowing for (**b**) Ultimet, (**c**) Ti6Al-4V, (**d**) AA2024, AA 2055 and AA6061 (**e**) 304 SS, 316 SS, AISI 1018 CS. AM 350 and Custom 450.

**Figure 3:**
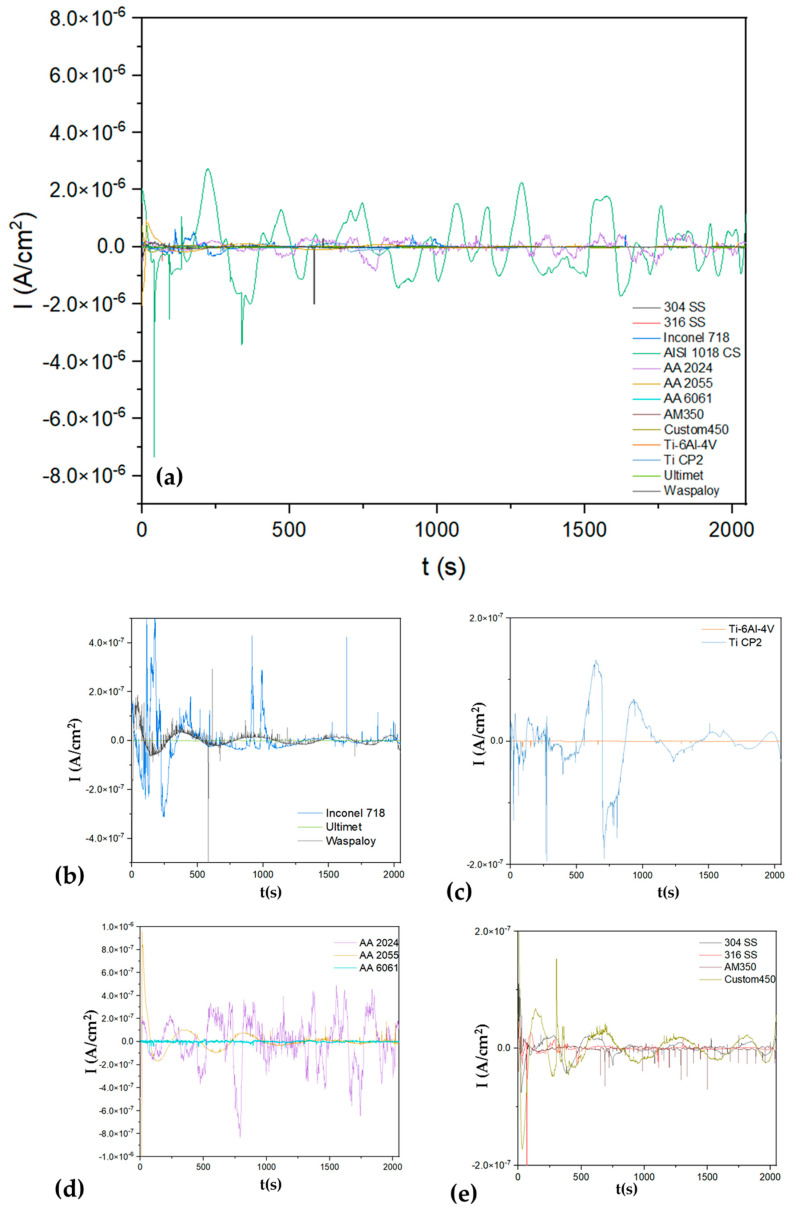
Electrochemical current noise-time series for alloys in NaCl (**a**) and windowing for (**b**) Inconel 718, Ultimet and Waspaloy, (**c**) Ti-6Al-4V and Ti CP2, (**d**) AA 2024, AA 2055 and AA 6061 (**e**) 304 SS, 316 SS, AM 350 and Custom 450.

**Figure 4:**
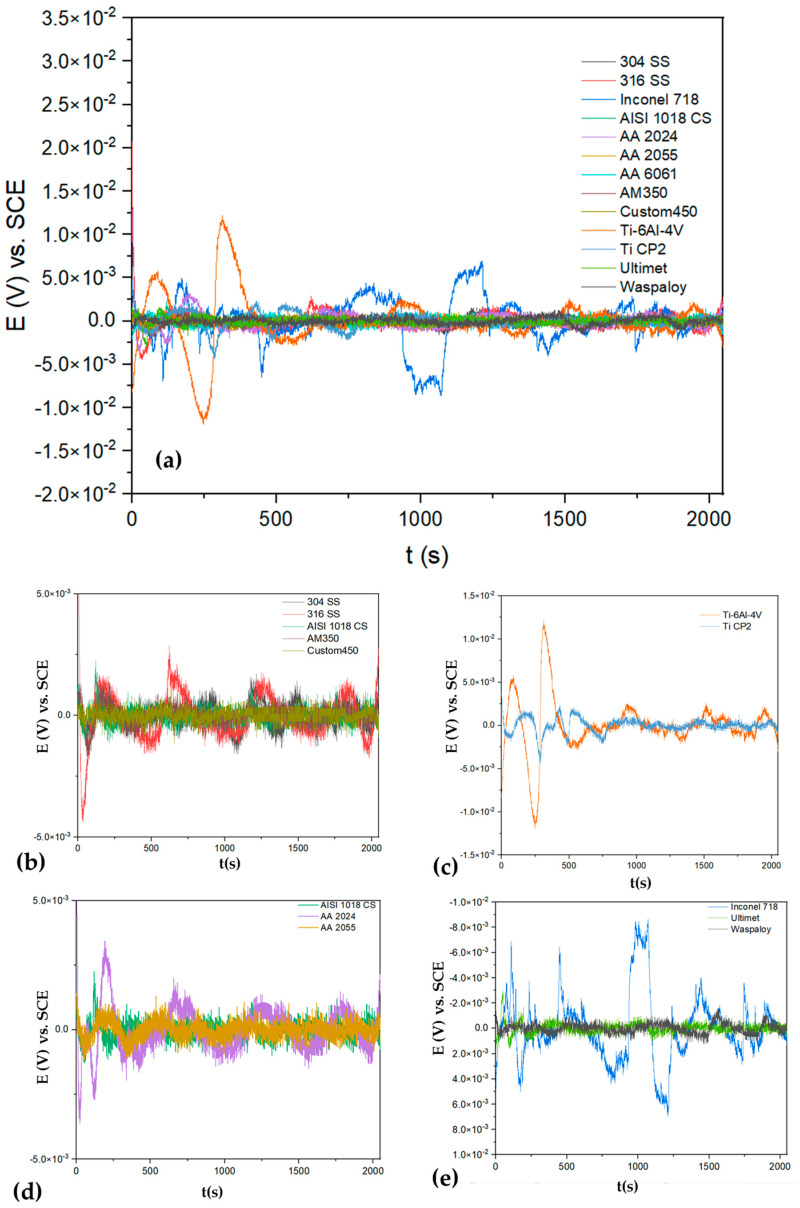
Electrochemical potential noise-time series for alloys in H_2_SO_4_ (**a**) and windowing for (**b**) 304 SS, 316 SS, AISI 1018 CS, AM 350 and Custom 450, (**c**) Ti-6Al-4V and Ti CP2, (**d**) AA2024, AA 2055 and AISI 1018 CS, (**e**) Inconel 718, Ultimet and Waspaloy.

**Figure 5:**
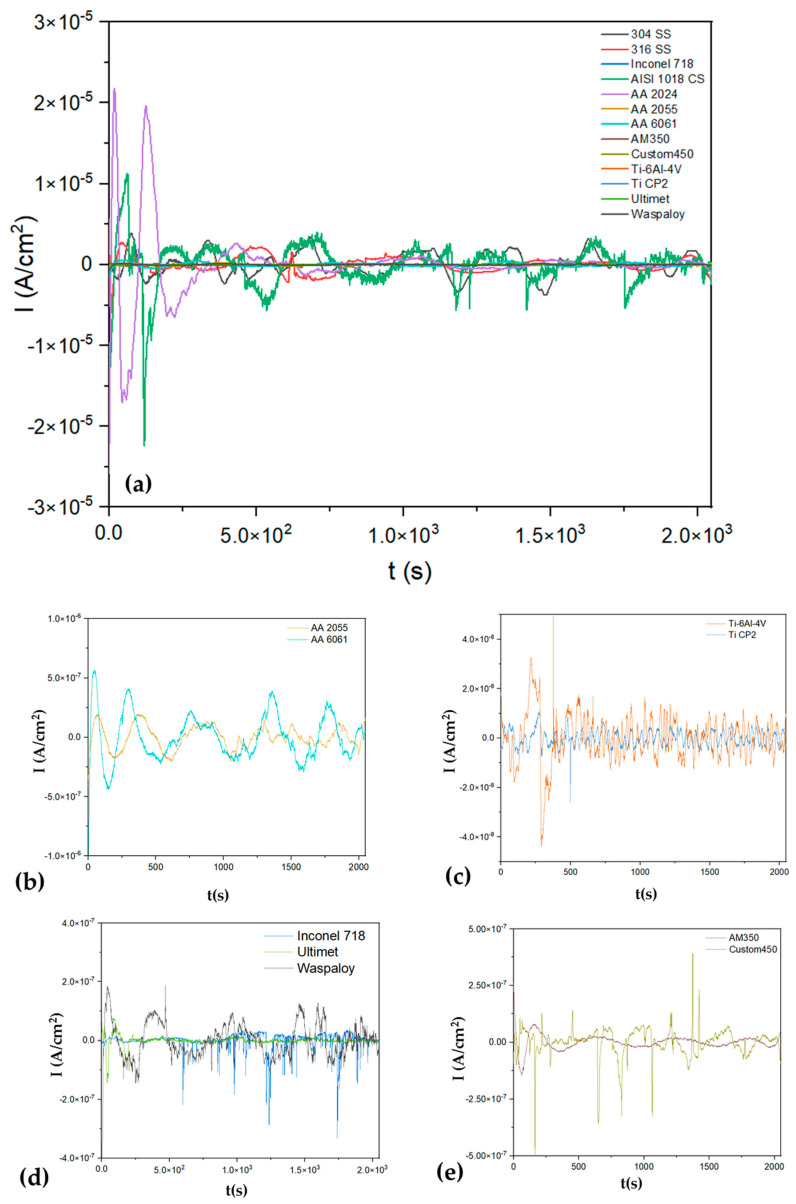
Electrochemical current noise-time series for alloys in H_2_SO_4_ (**a**) and windowing for (**b**) AA2024 and AA 2055, (**c**) Ti-6Al-4V and Ti CP2, (**d**) Inconel 718, Ultimet and Waspaloy (**e**) AM350 y custom450.

Figure 2, Figure 3, Figure 4 and Figure 5 shows the time series recorded for all the alloys’ potential and current. Figure 2a shows the electrochemical potential noise (EPN) when alloys were exposed to NaCl. Figure 2b shows the low amplitude and frequency transients for the Ultimete alloy; these transients are characteristic of generalized corrosion. The Ti-6Al-4V, Figure 2c, presented transients indicating the breaking and regeneration of the passive layer. On the other hand, AA 2024, Figure 2d, presented a higher potential amplitude of 4 × 10^−4^ V vs. SCE; those fluctuations are related to a mixed corrosion process. The behavior that presented steels, Figure 2e, is associated with a localized process that presented many transients with amplitudes.

Figure 3a shows the electrochemical current noise (ECN); it presents a high amplitude signal for AISI 1018 CS, indicating a possible higher corrosion kinetic with values of 6 × 10^−6^ A/cm^2^, and a cathodic transient of 8 × 10^6^ A/cm^2^, indicating a possible localization process. In Figure 3b, superalloys presented anodic transients (8 × 10^−7^ A/cm^2^), and Inconel 718 presented more of this behavior, associating with a localized process for this superalloy. On the other hand, Ultimet and Waspaloy presented a fluctuation with a transient system of low amplitude, relating that process to a possible passivation system. The Ti-6Al-4V presented in Figure 3c shows the behavior of a passive system with a very low signal. In the case of steels, all presented anodic transients of 1 × 10^−7^ A/cm^2,^ indicating that a localized process occurs on the surface. The aluminum alloys’ behavior differs for each (see Figure 3d); the AA 2024 presented high fluctuations, 1 × 10^−6^ A/cm^2^, indicating a higher corrosion kinetic mean, while the AA 6061 presented lower fluctuations, indicating low corrosion kinetic. Those results are shown in Table 1, where the AA 6061 presented 170,057 Ω·cm^2^ and the AA 2024 43,859 Ω·cm^2^.

Figure 4a shows the EPN of alloys exposed to H_2_SO_4_, and Figure 5a shows the ECN of alloys exposed to H_2_SO_4_. In almost all EPN alloys, Figure 5b–e presents a behavior related to the generation of a passive layer. For this reason, it is important to analyze Figure 5 of the ECN to determine if passivation occurs or if another process exists. Figure 4a shows how the AISI 1018 CS presented anodic transients of 2 × 10^−5^ A/cm^2^, indicating a possible localized process. Also, it presented the highest amplitude and AA 2024 (1 × 10^−4^ A/cm^2^), indicating that corrosion kinetics are higher (see Figure 5b). The results correspond to Table 2, where AISI 1018 CS and AA 2024 presented R_n_ values of 130 and 249 Ω·cm^2^. The signal of Ti-6Al-4V and Ti CP2 is related to a passive system (Figure 5c). On the other hand, Inconel 718 (Figure 5d) and Custom 450 (Figure 5e) observed anodic transients of 4 × 10^−7^ A/cm^2^, indicating a localized corrosion process.

Table 1 shows the statistical results, which will be correlated with the time-series analysis. The R_n_ shows a higher resistance for Ti-alloys (17.5 × 10^4^ and 20.4 × 10^4^ Ω·cm^2^); meanwhile, the AISI 1018 CS presented a lower corrosion resistance of 2.8 × 10^4^ Ω·cm^2^. Comparing the localization index in Table 3, any one of the samples presented a uniform process; all presented localized or mixed processes. When kurtosis analyzed samples, all samples presented localized corrosion in NaCl solutions. However, when analyzed by skewness, 304 SS, AISI 1018 CS, AA 2024, Ti-6Al-4V, and Ti CP2 presented uniform corrosion. This marks a difference in the analysis and generates uncertainty for the analysis.

For the samples analyzed on H_2_SO_4_, the results (see Table 2) were very similar; Ti-6Al-4V and Ti CP2 presented higher values of R_n_ (32.5 × 10^4^ and 27.1 × 10^4^ Ω·cm^2^), and the AISI 1018 and AA 2024 presented lower corrosion resistance (130, 249, and 326 Ω·cm^2^). When corrosion type is analyzed by localization index, only AA 2055 (0.09) presented uniform corrosion, and the rest of the samples presented mixed and localized corrosion. When those samples were analyzed by kurtosis, 304 SS and AA 2055 presented uniform corrosion. On the other hand, skewness showed that 304 SS, AA 2024, AA 2055, AM350, Ti-6Al-4V, and Ti CP2 presented values related to uniform corrosion. Therefore, it is necessary to validate the results with other analysis methods.

#### 3.1.2. Frequency-Domain Analysis

##### Power Spectral Density and Noise Impedance (Z_n_)

**Figure 6:**
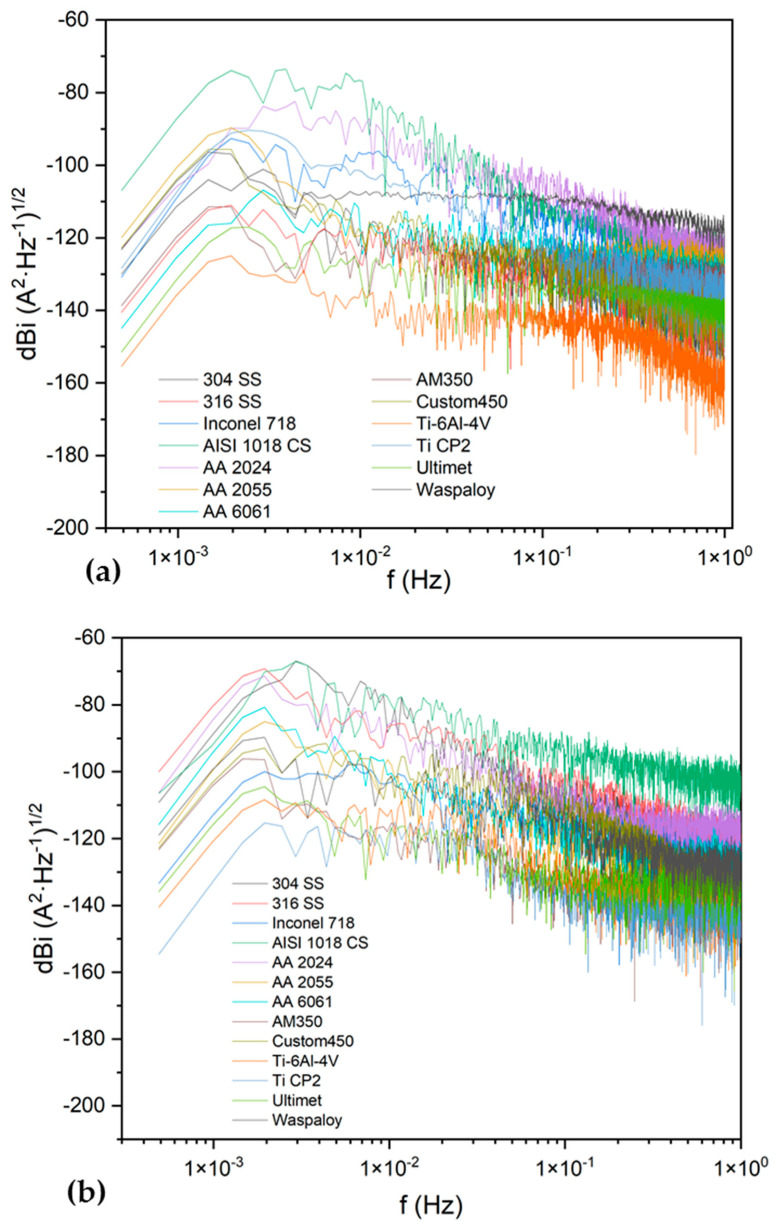
Power spectral density (PSD) in current for samples in NaCl (**a**) and H_2_SO_4_ (**b**).

**Figure 7:**
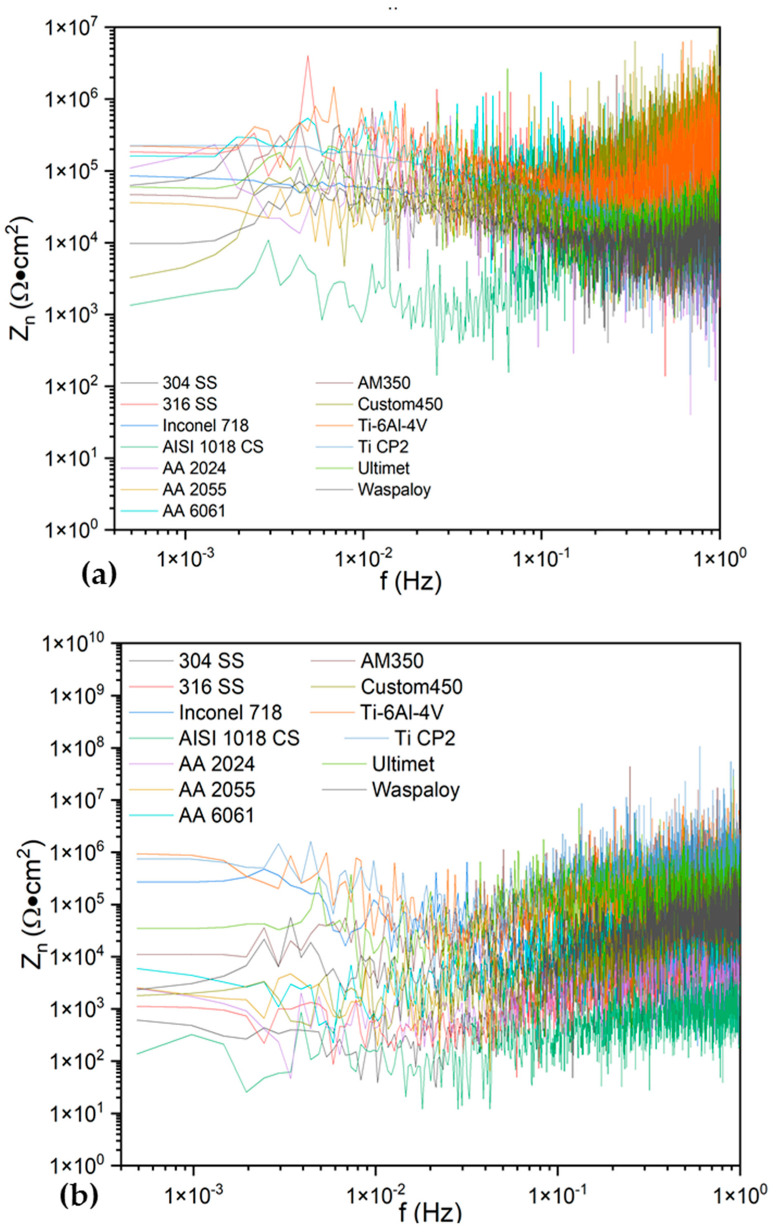
Noise impedance (Z_n_) for samples in NaCl (**a**) and H_2_SO_4_ (**b**).

Figure 6 and Figure 7 show the PSD and noise impedances of alloys. Table 3 presents the results of the parameters obtained in the frequency domain analysis. The AISI 1018 CS presented the lower Z_n_ (1.3 × 10^4^ Ω·cm^2^) in NaCl solution, followed by Custom 350, 304 SS, and 316 SS when exposed to NaCl and H_2_SO_4_ solutions. That behavior matches the results obtained by statistical analysis with the noise resistance (R_n_). The PSD slope results for alloys exposed to NaCl presented values of localized corrosion for SS 304, SS 316, Inconel 718, AISI 1018 CS, AA 2024, Ti-6Al-4V, and Ti CP2; the rest of the alloys presented values of uniform corrosion.

When the alloys were exposed to H_2_SO_4,_ only the Ultimet and AM350 alloys presented slope values of uniform corrosion; the rest of the samples presented localized corrosion according to the slope parameters. The AISI 1018 CS presented 136 Ω·cm^2^ of resistance, followed by the 304 SS with 612 Ω·cm^2^.

Limit frequency to cero showed results that were divergent with Z_n_; this indicated a variation between potential and current results. Some authors consider this to be correct. However, when the system is microbial, using the PSD in current as the direct parameter to determine corrosion kinetics is better; on the other hand, the slope shows values related to localization and uniform corrosion; however, those values do not match those obtained by statistical analysis.

#### 3.1.3. Time-Frequency Domain Analysis

##### Wavelets Analysis

Eight details and one estimate comprise the crystal numbers that need to be analyzed for this study. A metastable pitting process is thought to be responsible for energy accumulation on the first crystals (D1–D3). Localized corrosion is linked to major energy presented in crystals D4–D6, while diffusion, generalized, or controlled processes are thought to be responsible for energy in crystals D7 and D8 [24,30,35]. The DC from the EN signal is associated with the approximate crystal S8. Equation (15) [70] is employed to ascertain each crystal time range.

(15) $$\left(c_{1}^{j},c_{2}^{j}\right)=\left(2^{-j}\Delta t,2^{j-1}\Delta t\right)$$

where ∆*t* is the time display and *c* is the crystal. Every crystal scale ranges in both Hz and seconds. The initial crystals are high-frequency, whereas the latter display low-frequency phenomena.

**Figure 8:**
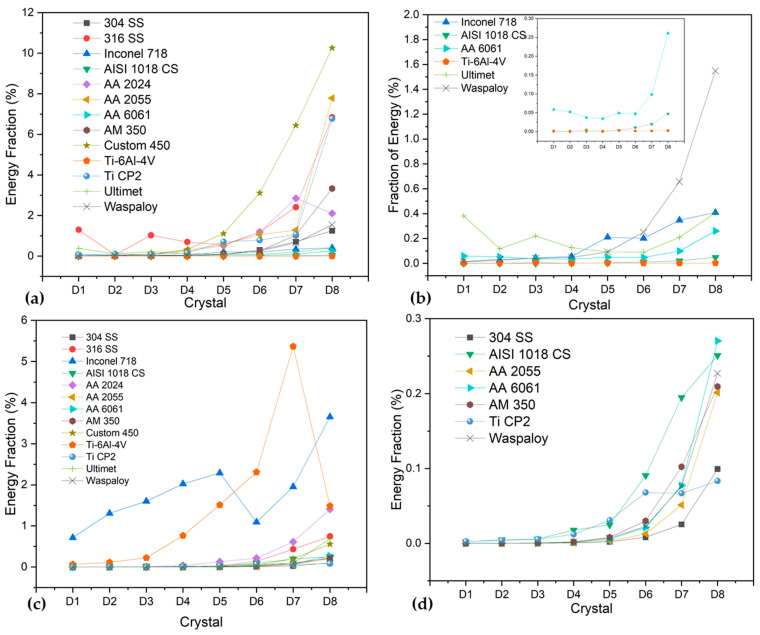
Energy dispersion plot in NaCl (**a**,**b**) and H_2_SO_4_ (**c**,**d**).

Figure 8 shows the energy dispersion plot calculated by the wavelet method. Figure 7a,b shows the results when exposed to NaCl solution. Figure 7 shows how almost all processes are dominated by uniform corrosion or diffusion. Only the Ti-6Al-4V and Ti- CP2 presented on passivation due to their low energy in the crystal. The 316 SS presented energy accumulation at the middle crystals, meaning a localized process; also, the energy at the last crystals presented more energy due to a pitting diffusion.

In Figure 8c,d, alloys were exposed to H_2_SO_4_. The AISI 1018 CS presented a similar behavior in H_2_SO_4_, where the pitting process is observed. The 316 SS is susceptible to pitting only in NaCl solutions.

##### Hilbert–Huang Transform Analysis and Recurrence Plots

**Figure 9:**
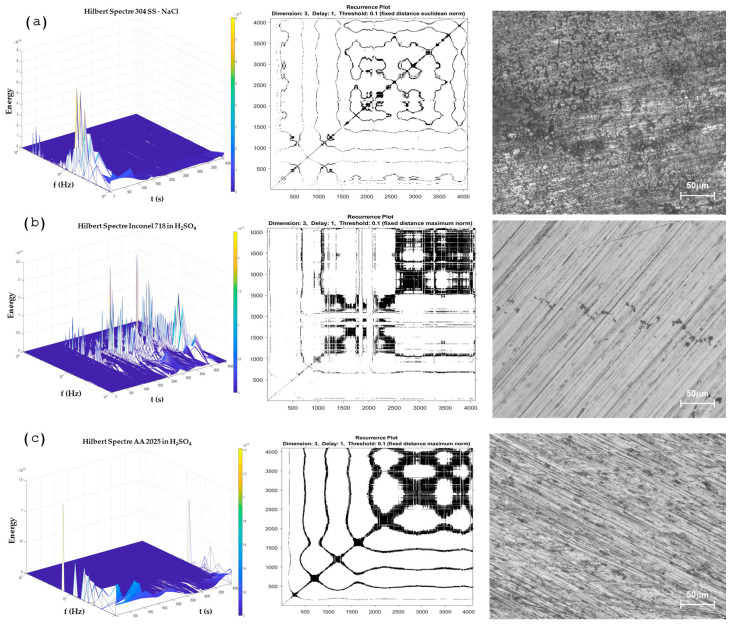
The Hilbert spectra with recurrence plot and morphology by optical microscopy. (**a**) 304 SS in NaCl, (**b**) Inconel 718 in H_2_SO_4_, (**c**) AA2025 in H_2_SO_4_.

Only the more significant samples were considered for this analysis due to the graphic number. Figure 9a,c shows a uniform corrosion process; the results obtained by the Hilbert specter and recurrence plot showed congruence, and the microscopy figure helps to show the congruence of the method. The HHT methods show energy accumulation at low frequencies, indicating that a long-term process is occurring. The RP shows a system with a difference in its repeatability, resulting in more recurrence. Also, the determinism value (DET) is higher than when samples present pitting (0.9856 and 0.9295). The value of AA 2025 is lower than the value of 304 SS due to the system being governed by pitting (uniform corrosion), and 304 SS is a uniform oxide layer created.

**Figure 10:**
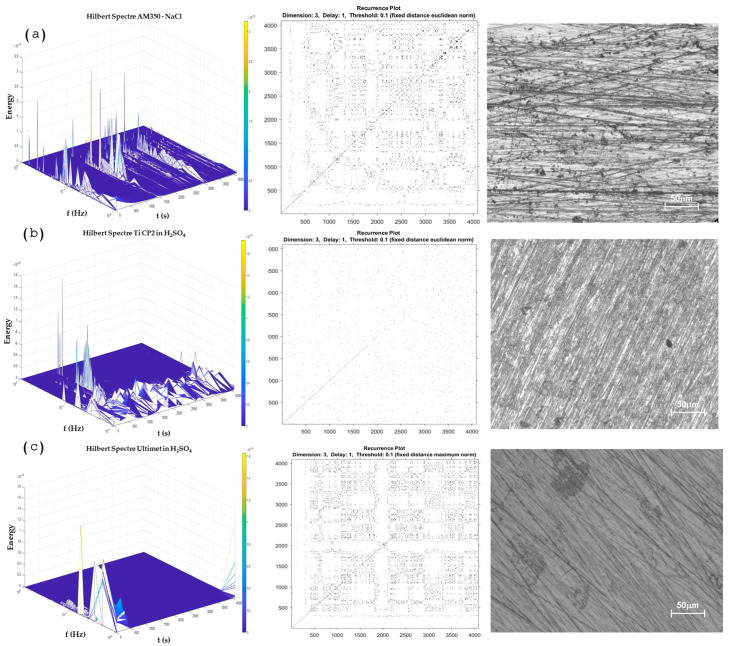
The Hilbert spectra with recurrence plot and morphology by optical microscopy. (**a**) AM350 in NaCl, (**b**) Ti CP2 in H_2_SO_4_, and (**c**) Ultimet in H_2_SO_4_.

On the other hand, Figure 9b and Figure 10a, shows a system domain by pitting, where the energy accumulation in the Hilbert specter showed energy at middle frequencies, and the RP presented a high presence of horizontal and vertical lines, indicating that a process is being repetitive; this occurs when localization is present on the surface. When it occurs, the determinist value is reduced, as shown in Table 4, where Inconel 718 obtained a value of 0.8402 and AM350 0.8022, lower than the values of more than 0.9 when alloys have a uniform corrosion system.

The passive system is present in Figure 10b,c; however, determinism results are lower (0.39 and 0.54 for Ti CP2 and Ultimet exposed to H_2_SO_4_). The behavior is sometimes confused with the pitting system due to the nature of how passivation occurs; however, the behavior of RP is more stochastic. The RP can present several differences between a passive and a localized system. The passive system presents dot morphology due to the passive system generating uniform micro-pitting attacks that generate the oxide layer. Therefore, the energy required is very low, and the vertical and horizontal lines are not formed as in the pitting system.

It is important to mention that alloys such as Ti-6Al-4V presented values of 0.7597 when exposed to H_2_SO_4_, indicating that the alloy tends to passivate.

## 4. Discussion

The statistical method showed that the type of corrosion does not correspond in almost all cases. This occurs due to the variability of LI; authors such as Eden and Mansfeld [4,9,71] mentioned that statistical analysis presents limitations, considering that Eden proposed the LI years before to establish the corrosion type. For this reason, LI should be used to determine the corrosion type at your discretion. Furthermore, the signal analyzed by the statistical method must not present a DC signal to reduce the standard deviation and present a more specific result [72]. In this research, the values obtained by LI do not converge with those obtained by skewness, kurtosis, wavelets, slope, HHT, and RPs in almost all results, creating confusion when analyzing the system. Only in steel has LI behavior presented some certainty; however, the limitations of this method are present.

Other statistical methods that are more certain are kurtosis and skewness. For those analyses, some results, such as 304 SS, reflect a result similar to that obtained by the HHT and RP analysie. However, skewness and kurtosis do not present a value for a passive system. The analysis by skewness is more exact than the kurtosis analyses. However, authors such as Cottis, Turgoose, Abella, Jaquez, and Sánchez-Amaya [10,11,72,73,74,75,76] recommend using the skewness with discretion due to statistical analysis’ limitations. Something similar occurred with kurtosis and skewness than in LI for this research, where the results between kurtosis and skewness do not match; meanwhile, kurtosis established a localized process, and skewness presented results of uniform corrosion in several alloys. This occurs due to the limitations of statistics and conventional methods for studying complex systems for corrosion. Furthermore, it is important to mention that the skewness results presented more certainty than kurtosis to determine a corrosion process in this research.

The results of PSD show the ψ^0^, Z_n,_ and slope to characterize the corrosion system. The slope analysis to determine the corrosion type presented values related to localized corrosion to 304 SS, 316 SS, Inconel 718, AISI 1018 CS, AA 2024, Ti-6Al-4V, and Ti CP2; the rest of the alloys presented uniform corrosion according to slope parameters in NaCl solution. In H_2_SO_4,_ only Ultimet presented a uniform corrosion value. The values of the slope parameters used to determine the corrosion type diverge from the results of the statistical method. That divergence is a parameter to consider in the slope analysis with some limitations; however, it is crucial to analyze the change at different PSD frequencies because this indicates changes in the corrosion process [77].

On the other hand, the values of Z_n_0 correspond to those obtained by R_n_, which shows that both methods are a good option for determining the corrosion resistance. The use of ψ^0^ to determine the corrosion kinetics is an option. However, in this case, it only helps to see what system is more active; some authors recommend using that value with a system where the measure of potential is compromised, such as microbial systems [78,79,80].

The wavelet analysis shows how almost all the samples in NaCl presented energy accumulation in the last crystals, indicating that the alloys presented a long corrosion process. Alloys such as Ti CP2 and Ti-6Al-4V presented low energy, indicating a passive system [81,82]. Several authors [41,42,43,44,56,57] mentioned that the wavelet method fits more with studying some dynamic systems. However, the results presented in this research present some limitations due to the necessity of studying a stationary signal; for that reason, more specialized methods must be implemented.

Methods such as HHT and RP present an excellent option. The HHT helps determine the type and process of corrosion that occurs when it is happening. Authors such as Homborg explain that the passive layer’s passivation, breaker, and regeneration can be observed in the Hilbert specter. HHT is considered one of the best options for analyzing some signals.

Wavelets and HHT have also been used for economic and mechanical analysis, biomedical industry, and corrosion applications. However, wavelet transform can be affected by the signal type and reconstruction, implying resolution limitations in the time and frequency domains. In contrast, HHT does not present those limitations due to the mathematical adaptation of the transform, allowing the analysis of non-stationary and stationary signals [83,84,85]. Hence, HHT is more relevant to the analysis of EN signals because it allows the study of many signals, reducing errors. Consequently, this research showed that HHT analysis presented good performance in characterizing the corrosion process in the system and the corrosion type. The results of HHT in this research presented the difference between a pitting and a uniform system. The pitting system presents a Hilbert specter with a high presence of energy at high and middle frequencies, and the uniform system presents energy at low frequencies. As suggested by these research authors, complementing the HHT analysis with the RPs is necessary. However, HHT does not present the limitations of statistical and slope methods.

In previous research, Calabrese et al. [34] concluded that data analysis by HHT is a better option to analyze EN in comparison with wavelets; in this research, that information was confirmed, and HHT is better than statistical PSD analysis to determine the corrosion process and type that occurs.

Several authors have used RP to determine corrosion type [51,74,75,76,77,78,83,84,85]. Valavanis et al. [86] suggest that RP can be used to determine dynamic states where transitions occur and physiochemically complex processes such as passivity, pitting, and uniform corrosion. Garcia-Ochoa [87] suggests that RP can be used to study non-linear systems and analyze the electrochemical process. Garcia-Ochoa concludes that RP and its quantifications are necessary for analyzing non-linear systems in different scientific disciplines. It is essential to mention that corrosion is a chaotic or non-linear system [88,89,90]. Due to those, the RP and RQA analyses presented values related to the corrosion process that occurs in the system. For that reason, the results showed that HHT and RP presented more congruence with the corrosion process that occurred on the alloy’s surface [91,92]. By this method, the difference between a pitting system (Figure 7a,b) and a passive system (Figure 8b,c) did not occur by conventional statistical methods or wavelets (in more complex systems). However, it is important to make a graphic analysis of RPs due to the values of determinism and recurrence (DET and RR), which presented similar values for a pitting-localized system than for a passive system. It occurred due to the passivation mechanism, propitiated by small pitting processes that generate the oxide layer. In DET and RR values, it cannot be observed and is nerveless; in RPs, a passive system is shown as a dot system, and a localized-pitting system is shown with vertical and horizontal lines that indicate the moment at which pitting occurs.

## 5. Conclusions

- This study concludes that statistical and slope (PSD) analysis present limitations in determining the corrosion type and some corrosion processes. This can be attributed to the complexity of the EN signal. It is the divergences that determine the type of corrosion that occurs in alloys.
- Statistical analysis presented limitations in all the analyses; localization index, kurtosis, and skewness showed different results in all the analyses. Some authors attribute this to the presence of different corrosion processes in the system; however, it can be confusing and speculative. For that reason, it is important to use a different method to determine the corrosion process in a material.
- The analysis of wavelets presented better results in determining corrosion type compared with statistical and PSD (slope) methods; however, the limitation of analyzing different types of signals limits this method.
- The analysis by HHT and RP presented the best results for determining the corrosion process and type. This is because methods present several advantages to analyzing chaotic signals. Hence, HHT and RP are recommended for analyzing EN signals more than statistical, wavelet, or slope methods. That is because of the nature of the corrosion signal; it is a complex signal, and the EN should be analyzed with the correct tools. The results converged with the results presented by the different authors mentioned in the discussion.
- It is important to complement the results of determinism and RR with the RPs as a visual reference. This is due to the results of DET and RR when a passive and a pitting system are presented. The pitting process presents DET values from 0.5 to 0.8 as well as a passivation system, but the graphic system is different. In passivation, a dot system is present, while in the pitting system, there are horizontal and vertical lines.
- In the HHT and RPs methods, the transition of corrosion processes as the break and regeneration of the passive layer, as well as the pitting generation of the predominance of a uniform process, can be observed. At HHT, the breaking of the passive layer is shown with energy at high frequencies and energy accumulation at low frequencies at subsequent seconds of energy presented at high frequencies.
- The use of R_n_ and Z_n_ to determine corrosion resistance can be accepted, and they presented similar results. The higher values were obtained by Ti C2 and Ti-6Al-4V exposed in H_2_SO_4_ with 271,851 and 325,751 Ω·cm^2^ by R_n_ and 742,824 and 939,575 Ω·cm^2^ by Z_n_. Although the values are not the same, R_n_ and Z_n_ can be considered homologues.
- It is important to define a method for correct EN analysis. If one analysis is correct, it can easily be applied to study neuronal networks or machine learning.
- EN is a powerful technique that can be employed in situ due to its non-destructive properties. Also, using this technique to detect different corrosion systems and some galvanic couples in the alloy phase is helpful for a correct alloy design.

## Figures and Tables

**Table 1:** Statistical parameters from time series of EN exposed to NaCl solution.

Alloys	R_n_ (Ω·cm^2^)	LI	Corrosion Type	Kurtosis	Corrosion Type	Skewness	Corrosion Type
304 SS	54,772 ± 11	0.07 ± 0.02	Mix	4.5 ± 0.8	Loc	−1 ± 0.2	Uni
316 SS	66,621 ± 10	0.2 ± 0.03	Loc	26 ± 2	Loc	−29 ± 0.8	Loc
Inconel 718	57,745 ± 8	0.12 ± 0.08	Loc	12 ± 1.2	Loc	1 ± 0.5	Loc
1018 CS	2817 ± 14	0.03 ± 0.01	Mix	4 ± 1.0	Loc	0 ± 0.7	Uni
AA 2024	43,856 ± 9	0.28 ± 0.1	Loc	4 ± 0.6	Loc	−1 ± 0.3	Uni
AA 2055	15,212 ± 20	0.18 ± 0.07	Loc	72 ± 3	Loc	−4 ± 0.8	Loc
AA 6061	170,057 ± 24	0.06 ± 0.02	Mix	22 ± 2.6	Loc	−2 ± 0.9	Loc
AM350	76,329 ± 13	0.02 ± 0.02	Mix	69 ± 4	Loc	−4 ± 1.2	Loc
Custom450	17,038 ± 24	0.16 ± 0.12	Loc	87 ± 6	Loc	5 ± 0.9	Loc
Ti-6Al-4V	175,051 ± 29	0.01 ± 0.01	Mixt	188 ± 5	Loc	−0 ± 0.3	Uni
Ti CP2	204,605 ± 24	0.36 ± 0.08	Loc	6 ± 1.5	Loc	−0 ± 0.6	Uni
Ultimet	75,342 ± 26	0.13 ± 0.03	Loc	538 ± 7	Loc	13 ± 1.1	Loc
Waspaloy	14,451 ± 14	0.05 ± 0.02	Mix	10 ± 1.3	Loc	−17 ± 1.4	Loc

**Table 2:** Statistical parameters from time series of EN exposed to H_2_SO_4_ solution.

Alloys	R_n_ (Ω·cm^2^)	LI	Corrosion Type	Kurtosis	Corrosion Type	Skewness	Corrosion Type
304 SS	326 ± 5	0.01 ± 0.02	Mix	3 ± 0.8	Uni	0 ± 0.2	Uni
316 SS	1208 ± 15	0.06 ± 0.02	Mix	6 ± 0.2	Loc	−0 ± 0.7	Uni
Inconel 718	90,112 ± 20	0.4 ± 0.1	Loc	32 ± 1.5	Loc	−4 ± 0.3	Loc
1018 CS	130 ± 8	0.06 ± 0.02	Mix	14 ± 1.1	Loc	−2 ± 0.1	Loc
AA 2024	249 ± 12	0.01 ± 0.01	Mix	15 ± 1.7	Loc	1 ± 0.1	Uni
AA 2055	3564 ± 29	0.009 ± 0.001	Uni	3 ± 1.3	Uni	−0 ± 0.3	Uni
AA 6061	2384 ± 20	0.4 ± 0.1	Loc	6 ± 0.8	Loc	−0 ± 0.3	Loc
AM350	12,563 ± 24	0.02 ± 0.004	Mix	14 ± 0.7	Loc	0 ± 0.6	Uni
Custom450	4484 ± 33	0.03 ± 0.02	Mix	15 ± 0.5	Loc	−2 ± 0.2	Loc
Ti-6Al-4V	325,751 ± 16	0.4 ± 0.02	Loc	6 ± 0.3	Loc	−0 ± 0.5	Uni
Ti CP2	271,851 ± 123	0.04 ± 0.005	Mix	4 ± 1.2	Loc	0 ± 0.2	Uni
Ultimet	25,560 ± 37	0.5 ± 0.04	Loc	28 ± 2.5	Loc	−3 ± 0.7	Loc
Waspaloy	6356 ± 57	0.01 ±	Mix	16 ± 1.1	Loc	−1 ± 0.6	Loc

**Table 3:** PSD parameters when alloys are exposed to NaCl and H_2_SO_4_ solutions.

NaCl Solution				H_2_SO_4_ Solution			
Alloys	Slope (dBi)	Limit Frequency	Z_n_ (Ω·cm^2^)	Alloys	Slope (dBi)	Limit Frequency	Z_n_ (Ω·cm^2^)
304 SS	−14 ± 1	−130 ± 12	63,123 ± 70	304 SS	−15 ± 1.2	−109 ± 5	612 ± 12
316 SS	−12 ± 0.8	−140 ± 15	183,756 ± 122	316 SS	−17 ± 1	−100 ± 3	1129 ± 67
Inconel 718	−13 ± 1.1	−130 ± 9	85,956 ± 130	Inconel 718	−10 ± 1.1	−133 ± 8	268,894 ± 50
1018 CS	−17 ± 1.2	−106 ± 14	1342 ± 27	1018 CS	−9 ± 0.5	−106 ± 4	136 ± 8
AA 2024	−15 ± 0.7	−122 ± 11	109,304 ± 159	AA 2024	−11 ± 0.4	−107 ± 8	2493 ± 36
AA 2055	−2 ± 0.5	−119 ± 8	36,459 ± 98	AA 2055	−15 ± 0.3	−121 ± 2	2567 ± 38
AA 6061	−6 ± 0.9	−145 ± 10	162,779 ± 211	AA 6061	−10 ± 0.7	−115 ± 9	5983 ± 69
AM350	−5 ± 0.3	−138 ± 14	46,864 ± 136	AM350	−7 ± 0.4	−123 ± 10	11,072 ± 96
Custom450	−2 ± 0.2	−123 ± 13	3242 ± 67	Custom450	−19 ± 1.3	−122 ± 9	1793 ± 82
Ti-6Al-4V	−12 ± 0.4	−155 ± 13	223,794 ± 177	Ti-6Al-4V	−12 ± 0.9	−140 ± 11	939,575 ± 241
Ti CP2	−13 ± 0.8	−128 ± 10	222,411 ± 389	Ti CP2	−9 ± 0.4	−154 ± 13	742,824 ± 265
Ultimet	−4 ± 0.5	−151 ± 17	59,992 ± 450	Ultimet	−4 ± 0.2	−135 ± 7	34,595 ± 76
Waspaloy	−5 ± 0.8	−123 ± 15	9656 ± 87	Waspaloy	−11 ± 0.3	−119 ± 9	2328 ± 53

**Table 4:** RP data obtained by quantitative analysis.

NaCl Solution				H_2_SO_4_ Solution			
Alloys	RR	Det	RR/Det	Alloy	RR	Det	RR/Det
304 SS	0.067 ± 0.002	0.985 ± 0.002	0.067 ± 0.002	304 SS	0.028 ± 0.002	0.953 ± 0.001	0.029 ± 0.002
316 SS	0.155 ± 0.03	0.989 ± 0.03	0.156 ± 0.03	316 SS	0.042 ± 0.004	0.970 ± 0.003	0.042 ± 0.003
Inconel 718	0.101 ± 0.07	0.975 ± 0.06	0.103 ± 0.07	Inconel 718	0.056 ± 0.003	0.802 ± 0.004	0.062 ± 0.004
1018 CS	0.042 ± 0.09	0.963 ± 0.07	0.044 ± 0.08	1018 CS	0.011 ± 0.003	0.642 ± 0.002	0.017 ± 0.003
AA 2024	0.017 ± 0.008	0.809 ± 0.007	0.021 ± 0.008	AA 2024	0.172 ± 0.004	0.990 ± 0.004	0.171 ± 0.004
AA 2055	0.171 ± 0.08	0.992 ± 0.06	0.173 ± 0.07	AA 2055	0.026 ± 0.004	0.800 ± 0.003	0.028 ± 0.003
AA 6061	0.001 ± 0.0002	0.269 ± 0.0001	0.004 ± 0.0002	AA 6061	0.025 ± 0.003	0.932 ± 0.003	0.030 ± 0.003
AM350	0.029 ± 0.005	0.802 ± 0.005	0.036 ± 0.005	AM350	0.058 ± 0.003	0.956 ± 0.001	0.060 ± 0.002
CUSTOM450	0.095 ± 0.002	0.990 ± 0.003	0.096 0.003	CUSTOM450	0.048 ± 0.007	0.958 ± 0.008	0.051 ± 0.008
Ti-6Al-4V	0.076 ± 0.001	0.960 ± 0.002	0.079 ± 0.002	Ti-6Al-4V	0.017 ± 0.004	0.759 ± 0.005	0.022 ± 0.005
Ti CP2	0.085 ± 0.007	0.986 ± 0.009	0.086 ± 0.008	Ti CP2	0.002 ± 0.003	0.393 ± 0.002	0.006 ± 0.003
Ultimet	0.039 ± 0.004	0.907 ± 0.003	0.043 ± 0.003	Ultimet	0.013 ± 0.007	0.544 ± 0.008	0.025 ± 0.008
Waspaloy	0.099 ± 0.002	0.967 ± 0.001	0.102 ± 0.001	Waspaloy	0.008 ± 0.001	0.064 ± 0.001	0.123 ± 0.001

## Data Availability

The data presented in this study are available on request from the corresponding author.
